# Effect of production quotas on economic and environmental values of growth rate and feed efficiency in sea cage fish farming

**DOI:** 10.1371/journal.pone.0173131

**Published:** 2017-03-13

**Authors:** M. Besson, I. J. M. de Boer, M. Vandeputte, J. A. M. van Arendonk, E. Quillet, H. Komen, J. Aubin

**Affiliations:** 1 Animal Breeding and Genomics Centre, Wageningen University, Wageningen, the Netherlands; 2 Génétique animale et biologie intégrative, INRA, AgroParisTech, Université Paris-Saclay, Jouy-en-Josas, France; 3 Animal Production Systems group, Wageningen University, Wageningen, the Netherlands; 4 IFREMER, Chemin de Maguelone, Palavas-les-Flots, France; 5 INRA, Agrocampus Ouest, UMR1069 Sol Agronomie Spatialisation, Rennes, France; Xiamen University, CHINA

## Abstract

In sea cage fish farming, production quotas aim to constrain the impact of fish farming on the surrounding ecosystem. It is unknown how these quotas affect economic profitability and environmental impact of genetic improvement. We combined bioeconomic modelling with life cycle assessment (LCA) to calculate the economic (EV) and environmental (ENV) values of thermal growth coefficient (TGC) and feed conversion ratio (FCR) of sea bass reared in sea cages, given four types of quota commonly used in Europe: annual production (Qprod), annual feed distributed (Qannual_feed), standing stock (Qstock), and daily feed distributed (Qdaily_feed). ENV were calculated for LCA impact categories climate change, eutrophication and acidification. ENV were expressed per ton of fish produced per year (ENV(fish)) and per farm per year (ENV(farm)). Results show that irrespective of quota used, EV of FCR as well as ENV(fish) and ENV(farm) were always positive, meaning that improving FCR increased profit and decreased environmental impacts. However, the EV and the ENV(fish) of TGC were positive only when quota was Qstock or Qdaily_feed. Moreover, the ENV(farm) of TGC was negative in Qstock and Qdaily_feed quotas, meaning that improving TGC increased the environmental impact of the farm. We conclude that Qstock quota and Qdaily_feed quota are economically favorable to a genetic improvement of TGC, a major trait for farmers. However, improving TGC increases the environmental impact of the farm. Improving FCR represents a good opportunity to balance out this increase but more information on its genetic background is needed to develop breeding programs improving FCR.

## Introduction

The production of fish in sea cages releases, without being filtered, nutrients to the surrounding environment. The accumulation of organic matter stemming from the cages on the benthos may cause eutrophication, which affects the natural ecosystem [1, 2]. In all European countries, therefore, producing fish in sea cages has to comply with regulatory measures to limit the environmental impact. These measures require an environmental impact study of biotic and abiotic change3s due to the farming process [3, 4]. The aim of this environment impact study is to explore how much fish can be produced based on the carrying capacity of the natural ecosystem. The estimations can be supported by modeling tools. In Norway, for example, the Modelling—On growing fish farms—Monitoring (MOM) [5–7] is legally required by the Directorate of Fisheries for site selection [8]. The environmental impact study determines the delivery of the farming authorization accompanied by prescriptions that set a specific quota to the farm. The nature of the quota varies across European countries. In France, Greece or Spain, quotas constrain the annual production of fish or the annual feed distributed per farm [9, 10], whereas in Denmark, the quota is based on annual emission of nitrogen [4]. In Ireland, the production is constrained by the density of fish [4]. In Norway, the production of salmon is limited by the standing biomass per license and per site [11]. The main goal of these quotas is to limit the environmental impact of the farm to an acceptable level.

Nevertheless, fish farming is growing due to an increasing demand for fish products; the challenge, therefore, is to reach the demand while constraining or reducing environmental impacts. So far, the reduction of environmental impacts have been achieved through improving farming technologies and practices. For instance, Ytrestøyl et al. [12] showed that salmon is now a net contributor of marine ingredients due to a lower inclusion of fish meal and dish oil in the feed. Genetic improvement could also be a tool to decrease environmental impacts as it acts at the source of emission by generating cumulative changes in animal performances [13, 14]. The economic (EV) and environmental value (ENV) of genetic improvement of traits included in the breeding goal can be estimated using bio-economic models. These values represent the economic or environmental impacts of a change in one trait keeping the other traits constant [15]. Environmental values were first calculated in dairy systems using the principles of life cycle assessment (LCA) [14, 16, 17]. In fish farming, we combined a bioeconomic model with an LCA to estimate the economic and environmental values of thermal growth coefficient (TGC) and feed conversion ratio (FCR) of African catfish (*Clarias gariepinus*) produced in a recirculating aquaculture system, RAS [18]. The results showed that in dairy as well as in fish farming, genetic change of a trait can simultaneously reduce environmental impacts per unit of products and increase economic farm profit by improving production efficiency or production level. An improved production efficiency decreases the amount of resources needed per unit of product. A higher production level dilutes fixed environmental impacts over more production. However, as we showed in previous studies [18, 19], the economic and environmental values depended on the factors limiting production, which in the case of RAS are fish rearing density and nitrogen treatment capacity. In sea cages, the limiting factor is, most of the time, the production quota. The variety of quotas applied in sea cage farming suggests that genetic improvement might lead to a variety of economic and environmental responses, depending on which quota is being applied.

This study aims to investigate how different types of quota affect the economic and environmental impacts of genetic improvement in sea cage system. Using our bioeconomic / LCA model, we calculated the economic and environmental values of TGC and FCR in a sea cage system producing sea bass. First, we express ENV as a change of environmental impacts per ton of fish produced. This functional unit emphases the change in environmental efficiency of producing fish after genetic improvement. However, in the context of quotas such a functional unit cannot describe the dynamics of environmental impacts at farm level. Therefore, we also calculate ENV at farm level.

## Material and methods

### Bioeconomic model

The bioeconomic model developed in R [20] was based on the model described in Besson et al. [19] and Besson et al. [21]. In the present study the model was adapted to estimate the production of sea bass (*Dicentrarchus labrax*) in a hypothetical sea cage farm (based on a real farms data from Gloria Maris) constrained by quota. The farm was composed of 34 circular cages of 600 m^3^ for pre-growing and 34 circular cages of 1,800 m^3^ for on-growing. Fish were stocked at 10 g and sold at a fixed harvest weight of 400 g. Stocking occurred all year round. The bioeconomic model was divided in four model parts.

1. The fish model estimates individual fish growth using the thermal growth coefficient (TGC) corrected for the concave relationship between growth rate and temperature [22]. The time to reach harvest weight, therefore, varied according to the daily temperature encountered by the fish and thus according to the stocking date. Feed conversion ratio (FCR) was modelled by combining a specific seabass model from Person-Le Ruyet et al. [23] with a model from [24]. The fish model also estimates the individual emission of nutrient based pollutants using mass-balance [25, 26]. Further details about the fish model are given in S1 Table.
2. The batch model estimates the average stocking density of a batch depending on individual fish performances (from fish model) and mortality. A batch is defined as the group of fish stocked at the same time in the same pre-growing cage.
3. The farm model estimates the number of batches produced to calculate annual fish production, emission of pollutants, and annual feed consumption. At farm level, a quota is applied which constrain farm production. In the reference scenario the production of the farm was set to 1,000 tons per year and four different quotas were tested:
  - Production quota (Qprod). The production of the farm was limited to 1,000 tons per year.
  - Feed quota (Qannual_feed). The total amount of feed distributed per year per farm was limited to 2,050 tons.
  - Standing stock (Qstock). The instant biomass present on site at any day of the year was constrained to 435 tons.
  - Daily feed distribution (Qdaily_feed). The amount of feed distributed per day per farm was limited to 4 tons.
  The values of each quota was set to allow the farm to produce 1,000 tons in the reference scenario. In this study, we considered that O_2_ availability was never limiting. In every quota scenario, the density of stocking was considered fixed along the year. The outputs of the bioeconomic model were used to generate inventory data for the LCA.
4. Finally, in the economic model, annual profit is calculated by combining results of the farm model with economic parameters.

### Life cycle assessment

#### Goal and scope

LCA is a standardized method conceived to calculate the environmental impact of a production chain, from raw material extraction up to the product's end-of life [27]. In this study, we applied LCA according to the main specifications of ILCD standards.

The system was defined from cradle-to-farm-gate and included five distinct sub-systems: (1) production of purchased feed, including production of ingredients, processing, and transportation; (2) production of energy expended at farm level (electricity, gas and petrol); (3) production of farming facilities and equipment; (4) Chemical used, including the production and the use of anti-fouling for nets; (5) farming operation, including nutrient based pollutants emission from biological transformation of feed.

The functional units in which environmental impacts were expressed was (a) per ton of fish produced on a basis of one year of routine production (impact_fish) and (b) per farm on a basis of one year of routine production (impact_farm). impact_fish and impact_farm were used to calculate environmental values.

#### Life cycle inventory

1. Production of purchased feed—Crop-derived ingredients originated from Brazil and France (e.g. soybean meal from Brazil and wheat from France), whereas fish-derived ingredients originated from the Peruvian fish milling industry (Biomar, pers com, 2014). The chemical composition of the diet and the origin of the ingredients are given in S2 Table. The exact composition is not given to respect confidentiality. Economic allocation was used to calculate the environmental impacts of processes yielding multiple products in the feed production industry [28]. Ingredients were transported to the feed manufacture in France (Aquitaine) by transoceanic ship and by lorry (>32t), whereas the transport of feed from feed mill to the fish farm in southern France was by lorry (>32t). Transport distances and other data required to compute the environmental impact of feed ingredients were based on Boissy et al. [29] and Pelletier and Tyedmers [30], presented in detail in S2 and S3 Tables.
2. Production of energy expended on farm—The energy consumed by the farm was considered fixed. The energy consumption was set at 25,000 l of diesel per year, 55,000 l of petrol per year and 110,000 kWh of electricity per year. The electricity used by the farm was coming from the French energy mix proposed by EcoInvent v3 database. Contribution analysis is available in S4 Table.
3. Production of farming facilities and equipment used—We considered the construction of a building of 650 m^2^ with a life span of 30 years. The equipment includes cages (64 in total), vehicles (2 boats and 6 barges, 2 trucks, 1 car), two ice making machines and other small equipment (i.e. plastic buckets). The use of building and equipment was considering fixed per year at farm level. The background processes stem from EcoInvent v3. Contribution analysis is available in S5 Table.
4. Chemical used—This sub-system includes the emission from the production and the use of anti-fouling for nets. We considered that the nets were treated every nine months with water-based anti-fouling with copper dioxide at 24%.
5. Farm operation—The farm operation sub-system includes the emission of pollutants from biological transformation of the feed distributed to the fish. The amount of nitrogen (N), phosphorus (P) and chemical oxygen demand (COD) of the dissolved organic matter excreted by the fish directly into the sea were calculated through the bioeconomic model. Contribution analysis is available in S6 Table.

#### Life cycle impact assessment

Each flow observed in the system was assigned to different impact categories relatively to its potential environmental effects. We chose to investigate eutrophication, acidification and climate change [27], because fish farming contributes most to these environmental impact categories [31, 32]. The characterization factors from CML2 Baseline 2000 version 2.04 were used for eutrophication and climate change. The impact categories were calculated using Simapro 7.0 software.

### Economic and environmental values

Economic (EV) and environmental values (ENV) represent the change in profit and environmental impacts due to genetic improvement in a trait, while keeping other traits in the breeding goal constant. We calculated EV and ENV for two important traits representing production performance of a farm: TGC and the FCR. EV and ENV were calculated for each quota scenario. The genetic improvement implemented was one genetic standard deviation (σg) from the mean (μ). μTGC = 2.25, σg-TGC = 0.23 [33] and μFCR = 2.0, σg-FCR = 0.38. The genetic standard deviation of FCR was calculated according to Sutherland [34] using data from Kause et al. [35]. EVs were calculated as the difference between the annual profits of the farm after genetic change minus before genetic change. We calculated EVs at farm scale because the aim of selecting breeding is to increase farmer’s income. Then, the EVs were rescaled per ton of fish for better visualization. This rescaling is done before genetic gain to account for the economic gain of an increase of production after genetic change [36]. The EV was expressed in euro per ton of fish produced:

$$\text{EV}=\frac{{\text{Profit}}_{\mu +\sigma_{\text{g}}}-{\text{Profit}}_{\mu }}{{\text{Production}}_{\mu }}$$ (1)

Environmental values were first calculated per ton of fish produced; ENV(fish). ENV(fish) were calculated as environmental impacts (category level) per ton of fish produced before genetic improvement minus environmental impacts per ton of fish produced after genetic improvement:

$$\text{ENV}\left(\text{fish}\right)=\text{Impact}\_{\text{fish}}_{\mu +\sigma_{\text{A}}}-\text{Impact}\_{\text{fish}}_{\mu }$$ (2)

ENV(fish) express, therefore, the capacity of a trait to improve environmental efficiency of fish production. However, the aim of the quota is mainly to limit the impact of the farming process on the local environment. Hence, we also calculated environmental values at farm level; ENV(farm). ENV(farm) estimate the capacity of a trait to affect environmental impacts of a specific farming site. They were calculated similarly as economic values i.e. difference between the impacts at farm level after genetic change minus the impacts at farm level before genetic change divided by annual production before genetic change. It means that the ENV(farm) were rescaled to impacts per farm per ton of fish to be able to compare with ENV(fish).

$$\text{ENV}\left(\text{farm}\right)=\frac{\text{Impact}\_{\text{farm}}_{\mu +\sigma_{\text{A}}}-\text{Impact}\_{\text{farm}}_{\mu }}{{\text{Production}}_{\mu }}$$ (3)

Each trait has an ENV(fish) and an ENV(farm) for each of the three impact categories investigated. ENV(fish) and ENV(farm) were also expressed in percentage of change.

$$\text{ENV}\left(\text{fish\%}\right)=\frac{\text{ENV}\left(\text{fish}\right)\times 100}{\text{Impact}\_{\text{fish}}_{\mu }}$$ (4)

$$\text{ENV}\left(\text{farm\%}\right)=\frac{\text{ENV}\left(\text{farm}\right)\times 100}{\text{Impact}\_{\text{farm}}_{\mu }}$$ (5)

## Results

Genetic improvement can affect the production level (i.e. tonnes of fish produced per year) and/or the production efficiency of the farm (i.e. quantity of input used per unit of fish produced) (Table 1). These changes affect the economic and environmental values of thermal growth coefficient (TGC) and feed conversion ratio (FCR).

**Table 1:** Summary of the consequences of genetic improvement in TGC (thermal growth coefficient) and FCR (feed conversion ratio) on technical performances of a sea cage farm constrained by different quota. Qprod is the quota on annual production, Qannual_feed is on annual feed distributed, Qstock is on the daily biomass present on site and Qdaily_feed is on daily feed distributed.

Quota type	Improving TGC	Improving FCR
Qprod	none	Production efficiency
Qannual_feed	none	Production + Production efficiency
Qstock	Production	Production efficiency
Qdaily_feed	Production	Production + production efficiency

### Economic values, EV (Tables 1 and 2)

**Table 2:** Effect of different values of Thermal Growth Coefficient (TGC) and Feed Conversion Ratio (FCR) on fish production parameters in different quota scenarios. Qprod is the quota on annual production, Qannual_feed is on annual feed distributed, Qstock is on the daily biomass present on site and Qdaily_feed is on daily feed distributed.

Quota	TGC	FCR	Days to reach harvest weight (d)	Number of batch produced (#)	Production per batch (t)	Production at farm (t)	Feed consumption per farm (t)	Incomes (€ × 1000)	Feed cost (€ × 1000)	Juveniles cost (€ × 1000)	Fixed cost (€ × 1000)	Profit (€)	EV TGC (€/kg)	EV FCR (€/kg)
Qprod	2.25	2.02	573	30.87	32.4	1000	2047	5598	2662	692	2245	-0.03	0	0.5
	2.48	2.02	528	35.64	28.1	1000	2047	5598	2662	692	2245	-521.88		
	2.25	1.64	573	30.87	32.4	1000	1660	5598	2159	692	2245	503048.8		
Qannual_feed	2.25	2.02	573	31.22	32.0	1000	2047	5597	2661	692	2245	0.56	0	1.14
	2.48	2.02	528	36.43	27.4	1000	2047	5597	2661	692	2245	-521.19		
	2.25	1.64	573	28.97	42.5	1232	2047	6900	2661	853	2245	1141326		
Qstock	2.25	2.02	573	32.02	31.2	1000	2046	5596	2659	692	2245	0.46	0.12	0.5
	2.48	2.02	528	34.49	30.6	1055	2160	5904	2809	730	2245	120354.6		
	2.25	1.64	573	32.02	31.2	1000	1657	5596	2155	692	2245	504751.8		
Qdaily_feed	2.25	2.02	573	31.12	32.1	1000	2046	5597	2660	692	2245	0.59	0.08	0.95
	2.48	2.02	528	35.06	29.5	1035	2118	5792	2753	716	2245	77713.2		
	2.25	1.64	573	29.34	39.6	1162	1929	6506	2508	804	2245	949212.7		

#### EV of TGC

Improving TGC decreased the time to reach harvest size and increased daily feed intake. In consequence, production should increase as more batches can be produced (increasing batch rotation). When the quota was on annual production (Qprod), higher batch rotation was balanced by lower stocking density to comply with the quota. This situation was similar with a quota on annual feed distributed (Qannual_feed). Thus, the economic value of TGC was null in Qprod and Qannual_feed. Conversely, when the quota was on daily standing stock (Qstock) or daily feed distributed (Qdaily_feed), increasing TGC led to more batches, but without a proportional decrease in stocking density. The resulting annual production was higher. Therefore, the economic value of TGC was positive: 0.12 €/kg of fish for Qstock and 0.08 €/kg of fish for Qdaily_feed.

#### EV of FCR

Improving FCR decreased the amount of feed required per unit of fish produced. When the quota was on feed distributed (Qannual_feed or Qdaily_feed) better FCR increased, therefore, production efficiency but production could also be increased until the feed quota was reached. Consequently, the economic value of FCR was 1.14 €/kg for Qannual_feed and 0.95 €/kg for Qdaily_feed. When Qprod or Qstock were the quotas, improving FCR improved only production efficiency. Thus, less feed was consumed at farm level and the economic value of FCR was 0.50 €/kg.

### Environmental value at fish level, ENV(fish)

More details about the results of the life cycle assessment are given in S7, S8 and S9 Tables.

#### ENV(fish) of TGC (Tables 3 and 4)

**Table 3:** Effect of different values of Thermal Growth Coefficient (TGC) and Feed Conversion Ratio (FCR) on annual emission of pollutants and environmental impacts in different quota scenarios. Qprod is the quota on annual production, Qannual_feed is on annual feed distributed, Qstock is on the daily biomass present on site and Qdaily_feed is on daily feed distributed.

Quota	TGC	FCR	Production per farm (t)	Nitrogen emission (t)	COD emission (t)	Phosphorus emission (t)	Climate change (kg CO_2_-eq / ton of fish)	Eutrophication (kg PO_4_-eq / ton of fish)	Acidification (kg SO_2_-eq / ton of fish)
Qprod	2.25	2.02	1000	114.92	2614.15	16.44	3636.53	168.62	21.77
	2.48	2.02	1000	114.98	2616.06	16.45	3636.53	168.73	21.77
	2.25	1.64	1000	88.29	1959.10	12.18	2995.38	127.90	18.33
Qannual_feed	2.25	2.02	1000	114.87	2613.11	16.43	3636.02	168.59	21.76
	2.48	2.02	1000	114.93	2615.01	16.45	3636.02	168.69	21.76
	2.25	1.64	1232	108.84	2414.94	15.02	2949.75	127.66	17.66
Qstock	2.25	2.02	1000	114.79	2611.13	16.42	3635.28	168.51	21.76
	2.48	2.02	1055	121.20	2757.49	17.34	3622.52	168.57	21.57
	2.25	1.64	1000	88.11	1954.71	12.16	2991.65	127.68	18.31
Qdaily_feed	2.25	2.02	1000	114.86	2612.80	16.43	3635.76	168.58	21.76
	2.48	2.02	1035	118.94	2706.36	17.02	3627.54	168.67	21.64
	2.25	1.64	1162	102.59	2276.24	14.16	2960.63	127.67	17.83

**Table 4:** Environmental Value (ENV) of TGC (thermal growth coefficient) at fish and farm level in different quota. Qprod is the quota on annual production, Qannual_feed is on annual feed distributed, Qstock is on the daily biomass present on site and Qdaily_feed is on daily feed distributed. Between brackets is the percentage a change in environmental impacts. A negative sign means that the environmental impact considered increased after genetic change.

Quota	TGC	FCR	ENV at farm level			ENV at fish level		
			Climate Change	Eutrophication	Acidification	Climate Change	Eutrophication	Acidification
			(kg CO_2_-eq)	(kg PO_4_-eq)	(kg SO_2_-eq)	(kg CO_2_-eq)	(kg PO_4-_eq)	(kg SO_2_-eq)
Qprod	2.25	2.02	0	-0.11	0	0	-0.11	0
	2.48	2.03	(0%)	(-0.06%)	(0%)	(0%)		(0%)
Qannual_feed	2.25	2.02	0	-0.11	0	0	-0.11	0
	2.48	2.03	(0%)	(-0.06%)	(0%)	(0%)	(-0.06%)	(0%)
Qstock	2.25	2.02	-186.84	-9.35	-1	12.76	-0.06	0.19
	2.48	2.03	(-5.14%)	(-5.55%)	(-4.61%)	(0.35%)	(-0.04%)	(0.86%)
Qdaily_feed	2.25	2.02	-118.74	-5.97	-0.63	8.22	-0.09	0.12
	2.48	2.03	(-3.25%)	(3.54%)	(-2.91%)	(0.23%)	(-0.05%)	(0.55%)

ENV(fish) of TGC were null for all impact categories when the quota was Qprod or Qannual_feed because in this situation TGC did not increase production or production efficiency. However, when the quota was Qstock or Qdaily_feed, improving TGC increased production. When production increased, the fixed environmental impacts are diluted over more fish produced. The fixed environmental impacts are the production of energy, the use of chemical and the production of equipment and infrastructure. These impacts represent 16.4% of acidification and 6.7% of climate change. Consequently, the environmental impact per ton of fish produced decreased and the ENV(fish) of TGC were positive for acidification and climate change categories. However, fixed environmental impacts represent less than 1% of eutrophication. The 99% remaining are caused by feed production and fish excretion which increased with higher production in Qstock and Qdaily_feed. Therefore, ENV(fish) of TGC for eutrophication is close to zero in Qstock and Qdaily_feed.

#### ENV(fish) of FCR (Tables 3 and 5)

**Table 5:** Environmental Value (ENV) of FCR (feed conversion ratio) at fish and farm level in different quota. Qprod is the quota on annual production, Qannual_feed is on annual feed distributed, Qstock is on the daily biomass present on site and Qdaily_feed is on daily feed distributed. Between brackets is the percentage a change in environmental impacts. A positive value means that the environmental impact considered decreased after genetic change.

Quota	TGC	FCR	ENV at farm level			ENV at fish level		
			Climate Change	Eutrophication	Acidification	Climate Change	Eutrophication	Acidification
			(kg CO_2_-eq)	(kg PO_4_-eq)	(kg SO_2_-eq)	(kg CO_2_-eq)	(kg PO_4_-eq)	(kg SO_2_-eq)
Qprod	2.25	2.02	641.15	40.72	3.44	641.15	40.72	3.44
	2.48	2.03	(17.63%)	(24.15%)	(15.80%)	(17.63%)	(24.15%)	(15.80%)
Qannual_feed	2.25	2.02	0	11.23	0	686.27	40.93	4.11
	2.48	2.03	(0%)	(6.66%)	(0%)	(18.87%)	(24.28%)	(18.87%)
Qstock	2.25	2.02	643.63	40.83	3.45	643.63	40.83	3.45
	2.48	2.03	(17.71%)	(24.23%)	(15.86%)	(17.71%)	(24.23%)	(15.86%)
Qdaily_feed	2.25	2.02	194.12	20.16	1.04	675.13	40.91	3.94
	2.48	2.03	(5.34%)	(11.96%)	(4.79%)	(18.57%)	(24.27%)	(18.09%)

The ENV(fish) of FCR in all quotas is positive, i.e. improving FCR decreases environmental impacts, because improving FCR decreased the amount of inputs need to produce one tonne of fish. The decrease was higher for eutrophication because eutrophication also includes the reduction in pollutants emission from the fish.

### Environmental values at farm level, ENV(farm)

#### ENV(fish) of TGC (Tables 3 and 4)

ENV(farm) of TGC was null or close to null for all impact categories when quota was Qprod or Qannual_feed because TGC did not increase production or production efficiency. When the quota was Qstock or Qdaily_feed, production increased as well as feed consumption and emission of pollutants, which are the main contributors to environmental impacts. Consequently, the ENV(farm) of TGC was negative for all impact categories, meaning that increasing TGC increased environmental impacts at farm level. The increase of environmental impacts was higher in Qstock quota than in Qdaily_feed.

#### ENV(fish) of FCR (Tables 3 and 5)

When the quota was Qprod or Qstock, improving FCR improved production efficiency. Therefore, less feed was consumed at farm level and the ENV(farm) of FCR were positive for all impacts categories, meaning that environmental impacts decreased. When the quota was Qannual_feed, improving FCR increased production efficiency and production, which kept annual consumption of feed constant. Consequently the ENV(farm) of FCR for climate change and acidification was null. However, with better FCR, less nutrient based pollutants were emitted. It resulted in a decrease in eutrophication by 6.66% because the nutrients-based pollutants emitted to water were considered to remain in water and thus they were contributing to eutrophication only. Thus, for Qannual_feed, the ENV(farm) of FCR for eutrophication was positive. When the quota was Qdaily_feed, improving FCR increased at the same time production efficiency and production but less feed was consumed at farm level. It resulted in a positive ENV(farm) for FCR. The ENV(fam) of FCR was also higher for eutrophication than other impact categories because improving FCR reduces the amount of pollutants emitted.

## Discussion

In sea cage farming, quotas are implemented to limit the environmental impacts of farm operations, such as the deposition of organic matter under the cages and the emission of dissolved nutrients in water, leading to eutrophication. In the present study, we investigated the effect of these quotas on economic and environmental responses to genetic improvement of sea bass. This is the first time the effect of quotas on genetic improvement of fish is tested. Economic values are used to maximize the economic response in the breeding goal. These values weigh the traits of a breeding goal according to their impacts on farm profit. They are specific to production systems according to the quota applied [37] or the environmental conditions [38]. When EVs differ from one production system to another, a single breeding program may not be enough to maximize economic response in all production systems [38]. It is important to remember that EVs and ENVs represent the economic and environmental change due to an improvement in one trait while keeping the other traits of the breeding goal constant. Therefore, EVs and ENVs are informative about the economic and environmental importance of a particular trait, but they do not fully predict the response to selection of a breeding program. This could be particularly the case if, in our case, FCR and TGC were genetically correlated. However, this is poorly documented in fish at the present time, and while some studies show an improvement of FCR when TGC is genetically improved [39] others show none [40, 41].

In sea cage farming system, EVs of TGC and FCR varied across quota scenarios. When the quota was standing stock (Qstock) or daily feed distributed (Qdaily_feed), both TGC and FCR had a positive EV, but when the quota was annual production (Qprod) and annual feed (Qannual_feed), only FCR had a positive EV. Thus, in Qprod and Qannual_feed, economic gain would be achieved only if FCR could be improved, either by direct selection or by a genetically correlated response to improvement of TGC, meaning that increasing TGC would decrease FCR. However, there are no practical ways to directly select for FCR in fish as it is difficult to measure this trait in individual fish. Moreover, the existence and the magnitude of the genetic correlation between FCR and TGC is still debated [35, 39–41]. Conversely, Qstock and Qdaily_feed had positive values for EV of TGC. Consequently these are the quotas that will generate farm profit from selective breeding on TGC, which is easily achieved in fish breeding programs [42]. Additionally, Qstock and Qdaily_feed have a large positive EV for FCR, which could promote the inclusion of this trait in future breeding programs if efficient selection methods for this trait are developed. The different EVs observed across quotas imply, however, that different breeding programs would be needed to optimize economic response in each quota system.

The concerns about environmental impacts of aquaculture are increasing and, in the future, the objective of breeding programs might also shift towards decreasing environmental impacts instead of maximizing economic profit. To do so, environmental values could be used in breeding programs to orient them towards the reduction of environmental impacts. In this study, the environmental values were calculated per ton of fish produced (ENV(fish)) and per farm (ENV(farm)).

We calculated ENV(fish) because the aim of selective breeding could be to produce a kg of product with the lowest environmental impact. This type of production-related functional unit has been used for evaluating the environmental impacts of genetic improvement in dairy farming [15, 18] as well as in fish farming in RAS [19]. In this latter study, we found a positive ENV(fish) of TGC for climate change and eutrophication because faster growing fish could increase production, which diluted fixed environmental impacts (i.e. energy use). In RAS, fixed environmental impacts represent a high proportion of the total impacts, i.e. 42.4% of acidification and 26.9% of climate change in the above-mentioned study. This is because RAS is a highly technological production system requiring a lot of energy and equipment [43]. In a sea cage system, we also observed the same trend when Qstock and Qdaily_feed were the quotas. Nevertheless, ENV(fish) of TGC for acidification and climate change were small and even null for eutrophication because in sea cage system, there are less fixed environmental impacts to dilute with higher production. Fixed environmental impacts represented only 6.7% of climate change, 16.4% of acidification and 0.8% of eutrophication. Hence, the ENV(fish) are very sensitive to the type of system and to the proportion of fixed environmental impacts. Regarding FCR, the ENV(fish) were always positive for all impact categories meaning that environmental impacts decreases per ton of fish produced. According to these results, genetic improvement of TGC or FCR affects environmental impacts per ton of fish produced similarly in all quotas. Therefore, a single breeding program using ENV(fish) of TGC and FCR would minimize environmental impacts per ton of fish produced in all quota scenarios. In Europe, the production of sea bass was 156,449 t in 2014 and the production was expected to be about 178,000 t in 2017, which represent a growth of 13.77% [44]. If nothing is done, the subsequent increase in eutrophication would be about 13.5% as 98.2% of eutrophication is caused by variable environmental impacts (feed production and nutrient excretion). Thus, if we consider the eutrophication to be 168.57 kg of PO_4-eq_ per t of fish produced, the eutrophication at European scale would increase from 26,373 to 29,933 t of PO_4-eq_. Keeping eutrophication constant with the same increase in production would require a 11.9% improvement of FCR over 3 years (1 generation), which could be achieved by a 20% selection pressure (i = 1.4) on FCR if its heritability is 0.17 as suggested in Kause et al. [35].

In addition, the increase in production could also increase environmental impacts of farming site and the role of quota system is to prevent this increase. Thus, we chose to calculate ENV(farm) to estimate the capacity of a trait to affect environmental impacts of a specific farming site in compliance with the aim of quota system. At farm level, mitigation does not exist anymore and an increase in production increases feed distribution and hence nitrogen emission. Therefore, the ENV(farm) of TGC are negative in Qstock and Qdaily_feed. It means that, in Qstock and Qdaily_feed, the environmental response to selection on TGC can be interpreted differently whether we look at farm level or at fish level. This has been shown in dairy where genetic improvement in milk yield would increase emission of CO_2_-eq. at herd level but reduce emission per kilogram of milk produced [17]. Regarding FCR, genetic improvement would always decrease environmental impacts at farm level in every quota scenario. However, the ENV(farm) of FCR in Qannual_feed and Qdaily_feed are lower than in Qprod and Qstock because improving FCR in Qannual_feed and Qdaily_feed increases production level together with production efficiency. The variation of ENV(farm) of TGC and FCR suggests that a single breeding program including ENV(farm) would not minimize environmental response in all quotas. It also suggests that breeding objectives using ENV(farm) and ENV(fish) would not lead to the same response. Therefore, the choice between ENV(farm) and ENV(fish) depends on the objectives of the breeding program.

In our model, the only limiting factor was one of the four production quota, which implied a constant stocking density through the time. This stocking density was calculated to reach the quota limitation without overtaking it. In the reference scenario (without genetic improvement) we considered that the oxygen availability was not limiting the number of fish stocked. However, in the scenario with genetic improvement, increasing TGC increases individual oxygen consumption due to higher feed intake and increasing FCR decreases individual oxygen consumption due to lower feed intake. Therefore, if you considered oxygen limitation, changing TGC and FCR could impact the number of fish stocked in cages to avoid hypoxia. In Besson et al. [21], we showed that the economic value of TGC, when oxygen was the limiting factor, was null when the average temperature was 18°C and increased with higher average temperatures. These results suggest that the ENV(fish) of TGC would also increase with higher temperature when oxygen is the limiting factor. However, ENV(farm) would decrease with higher temperature. Regarding FCR, both the EV and ENV(fish) would be positive because better FCR decreases feed intake and oxygen consumption which would increase production and increase production efficiency. However, ENV(farm) would also be positive be only due to better production efficiency.

The economic and environmental sustainability of fish farming is already a main target of fish breeders. Since the 70’s and the development of the first breeding program for salmon, new traits have been included in breeding goals such as disease resistance or filet yield beside growth rate [42]. Nevertheless, the lack of knowledge on the potential economic and environmental impacts of these traits could generate economic shortfall for farmers and increase their environmental impacts. The advantages of using EV or ENV to weigh the traits in a breeding program is that they maximize the economic gain or the environmental gain of a breeding program. However, it is not possible to combine EV and ENV in a common breeding objective as they are not expressed with the same unit. EV is in euros while ENV is, for instance, in CO2-eq. Consequently, the economic breeding objective may have adverse effects on the environment. In that case, environmental values could be calculated differently. van Middelaar et al. [14] suggested to estimate the ENV of a trait using linear programming to minimize environmental response while keeping profit constant after genetic change.

## Conclusion

This is the first study investigating the influence of different quotas on the economic and environmental value of production traits in a selective breeding program. The results show that the economic and environmental responses change across quota scenarios, which suggest that each quota might need a specific breeding program in order to maximize profit or to minimize environmental impacts. It also suggests that policy makers could choose the quota depending on the objectives to achieve. For instance, standing stock quota and daily feed quota are economically favorable to a genetic improvement of growth rate, a major trait for farmers. However, in these quotas, improving growth increases the environmental impact of the farm. Improving FCR represents a good opportunity to balance out this increase in environmental impacts but we need more information on its genetic background to develop breeding programs including FCR. FCR is the most important trait to increase economic profit and to decrease environmental impacts in all scenarios.

## Supporting information

S1 Table Calculations and parameters involved in the fish model. (DOCX)

S2 Table Chemical composition of the feed of sea bass (Biomar, EFICO). (DOCX)

S3 Table Contribution analysis of 1 t of standard sea bass feed (Biomar, EFICO). (DOCX)

S4 Table Contribution analysis of energy carriers to acidification, eutrophication, and climate change. (DOCX)

S5 Table Environmental impacts of the construction of 1 m2y of buildings and of the production of all equipment needed at farm level. (DOCX)

S6 Table Environmental impacts of the emission to water of one ton of Nitrogen (N), Phosphorus (P) and Chemical Oxygen Demand (COD). (DOCX)

S7 Table Climate change per ton of fish produced for the five sub-systems as a function of Thermal Growth Coefficient (TGC) and Feed Conversion Ratio (FCR). Qprod is the quota on annual production, Qannual_feed is on annual feed distributed, Qstock is on the daily biomass present on site and Qdaily_feed is on daily feed distributed.
(DOCX)

S8 Table Eutrophication per ton of fish produced for the five sub-systems as a function of Thermal Growth Coefficient (TGC) and Feed Conversion Ratio (FCR). Qprod is the quota on annual production, Qannual_feed is on annual feed distributed, Qstock is on the daily biomass present on site and Qdaily_feed is on daily feed distributed.
(DOCX)

S9 Table Acidification per ton of fish produced for the five sub-systems as a function of Thermal Growth Coefficient (TGC) and Feed Conversion Ratio (FCR). Qprod is the quota on annual production, Qannual_feed is on annual feed distributed, Qstock is on the daily biomass present on site and Qdaily_feed is on daily feed distributed.
(DOCX)
