# Supplementary material for: Effect of production quotas on economic and environmental values of growth rate and feed efficiency in sea cage fish farming
Source: PLoS One. 2017 Mar 13;12(3):e0173131. doi: 10.1371/journal.pone.0173131 (PMC5347995; doi:10.1371/journal.pone.0173131)
Supplement: S1 Table — (DOCX) [file pone.0173131.s001.docx]

**S1 Table. Calculations and parameters involved in the fish model.**

| Parameters of fish model | Formulas |
| --- | --- |
|  |  |
| Thermal growth coefficient (TGC) :  *1-b = weight exponent = 0.51*  *K_i_ = daily corrected temperature*  *W_H_ (harvest weight) = 13 g*  *W_I_ ( initial weight) = 1300 g*  *n is the length of growing period until harvest weight* | $TGC =\frac{W_{H}^{1-b} - W_{I}^{1-b}}{\sum_{i=1}^{n} K_{i}}$ |
| Fish weight (W_n_) in kg : | ${W_{n}= [W_{I}^{0.51} + (TGC \times\sum_{i=1}^{n} K_{i})]}^{1/0.51}$ |
| Daily weight gain (DWG_n_) in g : | $\mathrm{DWG}_{n} = W_{n} - W_{n-1}$ |
| Feed conversion ratio (FCR_Wn_) in g/g : |  |
| $\mathbf{FCR}_{\mathbf{Wn}}\boldsymbol{= \alpha\times}\frac{\mathbf{W}_{\mathbf{n}}^{\mathbf{0.14}}}{\mathbf{1.318-}\left( \boldsymbol{0.103\times}\mathbf{T}_{\mathbf{i}} \right)\mathbf{+}\left( \boldsymbol{0.007174\times}{\mathbf{T}_{\mathbf{i}}}^{\mathbf{2}} \right)\boldsymbol{-(0.0001395\times}{\mathbf{T}_{\mathbf{i}}}^{\mathbf{3}}\mathbf{)}}$ | |
| Daily feed intake (DFI_n_) in g : | $\mathrm{DFI}_{n} = \mathrm{DWG}_{n} \times\mathrm{FCR}_{\mathrm{Wn}}$ |
