# Supplementary material for: Effect of production quotas on economic and environmental values of growth rate and feed efficiency in sea cage fish farming
Source: PLoS One. 2017 Mar 13;12(3):e0173131. doi: 10.1371/journal.pone.0173131 (PMC5347995; doi:10.1371/journal.pone.0173131)
Supplement: S2 Table — (DOCX) [file pone.0173131.s002.docx]

**S2 Table. Chemical composition of the feed of sea bass (Biomar, EFICO).**

| Chemical composition | % |
| --- | --- |
| Protein | 43 |
| Crude fat | 21 |
| Crude ash | 7 |
| Other Carbohydrates | 20 |
| Phosphorus | 1.1 |
| Components | Origin |
| Fish meal | Peru |
| Fish oil | Peru |
| Corn gluten | France |
| Rape meal | France |
| Rape oil | France |
| Soybean meal | Brazil |
| Wheat gluten | France |
| Wheat | France |
| Sunflower meal | France |
| Additive premix | France |
