# Supplementary material for: Effect of production quotas on economic and environmental values of growth rate and feed efficiency in sea cage fish farming
Source: PLoS One. 2017 Mar 13;12(3):e0173131. doi: 10.1371/journal.pone.0173131 (PMC5347995; doi:10.1371/journal.pone.0173131)
Supplement: S3 Table — (DOCX) [file pone.0173131.s003.docx]

**S3 Table. Contribution analysis of 1 t of standard sea bass feed (Biomar, EFICO).**

|  | Climate change,  kg CO_2_-eq | Eutrophication, kg PO_4_-eq | Acidification,  kg SO_2_-eq |
| --- | --- | --- | --- |
| Fish meal and oil | 22.02 % | 9.30 % | 19.70 % |
| Crops | 41.65 % | 82.26 % | 51.29 % |
| Other | 0.17 % | 0.35 % | 0.13 % |
| Feed processing, packaging and transportation | 36.16 % | 8.09 % | 28.87 % |
| Total % | 100 % | 100 % | 100 % |
| Total quantity | 1656.89 | 5.42 | 8.88 |
