# Supplementary material for: Effect of production quotas on economic and environmental values of growth rate and feed efficiency in sea cage fish farming
Source: PLoS One. 2017 Mar 13;12(3):e0173131. doi: 10.1371/journal.pone.0173131 (PMC5347995; doi:10.1371/journal.pone.0173131)
Supplement: S4 Table — (DOCX) [file pone.0173131.s004.docx]

**S4 Table. Contribution analysis of energy carriers to acidification, eutrophication, and climate change.**

|  | Climate change  (kg CO_2_-eq) | Eutrophication  (kg PO_4_-eq) | Acidification  (kg SO_2_-eq) |
| --- | --- | --- | --- |
| Electricity mix production (1000 kWh) | 94.35 | 0.18 | 0.53 |
| Diesel production (1000 l) | 532.71 | 0.80 | 5.11 |
| Fuel production (1000 l) | 518.02 | 0.75 | 5.16 |
