# Supplementary material for: Effect of production quotas on economic and environmental values of growth rate and feed efficiency in sea cage fish farming
Source: PLoS One. 2017 Mar 13;12(3):e0173131. doi: 10.1371/journal.pone.0173131 (PMC5347995; doi:10.1371/journal.pone.0173131)
Supplement: S5 Table — (DOCX) [file pone.0173131.s005.docx]

**S5 Table. Environmental impacts of the construction of 1 m2y of buildings and of the production of all equipment needed at farm level.**

|  | | Climate change  (kg CO_2_-eq) | Eutrophication  (kg PO_4_-eq) | Acidification  (kg SO_2_-eq) |
| --- | --- | --- | --- | --- |
| Construction of 1000 m^2^y of facilities | 9,586.57 | | 13.79 | 37.29 |
|  | |  |  |  |
| Production of total equipment used | | 27,300.7 | 59.57 | 146.95 |
|  | |  |  |  |
