# Supplementary material for: Effect of production quotas on economic and environmental values of growth rate and feed efficiency in sea cage fish farming
Source: PLoS One. 2017 Mar 13;12(3):e0173131. doi: 10.1371/journal.pone.0173131 (PMC5347995; doi:10.1371/journal.pone.0173131)
Supplement: S6 Table — (DOCX) [file pone.0173131.s006.docx]

**S6 Table. Environmental impacts of the emission to water of one ton of nitrogen (N), phosphorus (P) and chemical oxygen demand (COD).**

|  | | Climate change  (kg CO_2_-eq) | Eutrophication  (kg PO_4_-eq) | Acidification  (kg SO_2_-eq) |
| --- | --- | --- | --- | --- |
| 1 ton of N | 0 | | 0.42 | 0 |
| 1 ton of P | | 0 | 3.06 | 0 |
| 1 ton of COD | | 0 | 02 | 0 |
