# Supplementary material for: Effect of production quotas on economic and environmental values of growth rate and feed efficiency in sea cage fish farming
Source: PLoS One. 2017 Mar 13;12(3):e0173131. doi: 10.1371/journal.pone.0173131 (PMC5347995; doi:10.1371/journal.pone.0173131)
Supplement: S7 Table — Qprod is the quota on annual production, Qannual_feed is on annual feed distributed, Qstock is on the daily biomass present on site and Qdaily_feed is on daily feed distributed. (DOCX) [file pone.0173131.s007.docx]

**S7 Table. Climate change per ton of fish produced for the five sub-systems as a function of thermal growth coefficient (TGC) and feed conversion ratio (FCR). Qprod is the quota on annual production, Qannual_feed is on annual feed distributed, Qstock is on the daily biomass present on site and Qdaily_feed is on daily feed distributed.**

|  |  |  | Climate change (kg CO_2_-eq) / ton of fish) | | | | |
| --- | --- | --- | --- | --- | --- | --- | --- |
| Quota | TGC | FCR | Feed production | Energy use | Equipment and facilities | Chemical used | Farm operation |
| Qprod | 2.25 | 2.02 | 3392.34 | 52.14 | 110.76 | 81.29 | 0 |
|  | 2.33 | 2.02 | 3392.34 | 52.14 | 110.76 | 81.29 | 0 |
|  | 2.25 | 1.64 | 2751.19 | 52.14 | 110.76 | 81.29 | 0 |
|  |  |  |  |  |  |  |  |
| Qannual_feed | 2.33 | 2.02 | 3391.79 | 52.15 | 110.78 | 81.31 | 0 |
|  | 2.25 | 2.02 | 3391.79 | 52.15 | 110.78 | 81.31 | 0 |
|  | 2.33 | 1.64 | 2751.61 | 42.31 | 89.87 | 65.96 | 0 |
|  |  |  |  |  |  |  |  |
| Qstock | 2.25 | 2.02 | 3390.97 | 52.17 | 110.81 | 81.33 | 0 |
|  | 2.33 | 2.02 | 3390.97 | 49.44 | 105.02 | 77.08 | 0 |
|  | 2.25 | 1.64 | 2747.34 | 52.17 | 110.81 | 81.33 | 0 |
|  |  |  |  |  |  |  |  |
| Qdaily_feed | 2.25 | 2.02 | 3391.50 | 52.16 | 110.79 | 81.31 | 0 |
|  | 2.33 | 2.02 | 3391.50 | 50.40 | 107.06 | 78.58 | 0 |
|  | 2.25 | 1.64 | 2750.51 | 44.87 | 95.30 | 69.95 | 0 |
