# Supplementary material for: Effect of production quotas on economic and environmental values of growth rate and feed efficiency in sea cage fish farming
Source: PLoS One. 2017 Mar 13;12(3):e0173131. doi: 10.1371/journal.pone.0173131 (PMC5347995; doi:10.1371/journal.pone.0173131)
Supplement: S8 Table — Qprod is the quota on annual production, Qannual_feed is on annual feed distributed, Qstock is on the daily biomass present on site and Qdaily_feed is on daily feed distributed. (DOCX) [file pone.0173131.s008.docx]

**S8 Table. Eutrophication per ton of fish produced for the five sub-systems as a function of thermal growth coefficient (TGC) and feed conversion ratio (FCR). Qprod is the quota on annual production, Qannual_feed is on annual feed distributed, Qstock is on the daily biomass present on site and Qdaily_feed is on daily feed distributed.**

|  |  |  | Eutrophication (kg PO_4_-eq) / ton of fish) | | | | |
| --- | --- | --- | --- | --- | --- | --- | --- |
| Quota | TGC | FCR | Feed production | Energy use | Equipment and facilities | Chemical used | Farm operation |
| Qprod | 2.25 | 2.02 | 11.13 | 0.08 | 0.16 | 1.16 | 156.09 |
|  | 2.33 | 2.02 | 11.13 | 0.08 | 0.16 | 1.16 | 156.19 |
|  | 2.25 | 1.64 | 9.03 | 0.08 | 0.16 | 1.16 | 117.47 |
|  |  |  |  |  |  |  |  |
| Qannual_feed | 2.33 | 2.02 | 11.13 | 0.08 | 0.16 | 1.16 | 156.05 |
|  | 2.25 | 2.02 | 11.13 | 0.08 | 0.16 | 1.16 | 156.16 |
|  | 2.33 | 1.64 | 9.03 | 0.07 | 0.13 | 0.94 | 117.49 |
|  |  |  |  |  |  |  |  |
| Qstock | 2.25 | 2.02 | 11.13 | 0.08 | 0.16 | 1.16 | 155.98 |
|  | 2.33 | 2.02 | 11.13 | 0.08 | 0.15 | 1.10 | 156.12 |
|  | 2.25 | 1.64 | 9.02 | 0.08 | 0.16 | 1.16 | 117.26 |
|  |  |  |  |  |  |  |  |
| Qdaily_feed | 2.25 | 2.02 | 11.13 | 0.08 | 0.16 | 1.16 | 156.05 |
|  | 2.33 | 2.02 | 11.13 | 0.08 | 0.16 | 1.12 | 156.19 |
|  | 2.25 | 1.64 | 9.03 | 0.07 | 0.14 | 1.00 | 117.44 |
