# Supplementary material for: Effect of production quotas on economic and environmental values of growth rate and feed efficiency in sea cage fish farming
Source: PLoS One. 2017 Mar 13;12(3):e0173131. doi: 10.1371/journal.pone.0173131 (PMC5347995; doi:10.1371/journal.pone.0173131)
Supplement: S9 Table — Qprod is the quota on annual production, Qannual_feed is on annual feed distributed, Qstock is on the daily biomass present on site and Qdaily_feed is on daily feed distributed. (DOCX) [file pone.0173131.s009.docx]

**S9 Table. Acidification per ton of fish produced for the five sub-systems as a function of thermal growth coefficient (TGC) and feed conversion ratio (FCR). Qprod is the quota on annual production, Qannual_feed is on annual feed distributed, Qstock is on the daily biomass present on site and Qdaily_feed is on daily feed distributed.**

|  |  |  | Acidification (kg SO_2_-eq) / ton of fish) | | | | |
| --- | --- | --- | --- | --- | --- | --- | --- |
| Quota | TGC | FCR | Feed production | Energy use | Equipment and facilities | Chemical used | Farm operation |
| Qprod | 2.25 | 2.02 | 18.19 | 0.62 | 0.44 | 2.52 | 0 |
|  | 2.33 | 2.02 | 18.19 | 0.62 | 0.44 | 2.52 | 0 |
|  | 2.25 | 1.64 | 14.76 | 0.62 | 0.44 | 2.52 | 0 |
|  |  |  |  |  |  |  |  |
| Qannual_feed | 2.33 | 2.02 | 18.19 | 0.62 | 0.44 | 2.52 | 0 |
|  | 2.25 | 2.02 | 18.19 | 0.62 | 0.44 | 2.52 | 0 |
|  | 2.33 | 1.64 | 14.76 | 0.50 | 0.36 | 2.04 | 0 |
|  |  |  |  |  |  |  |  |
| Qstock | 2.25 | 2.02 | 18.19 | 0.62 | 0.44 | 2.52 | 0 |
|  | 2.33 | 2.02 | 18.19 | 0.58 | 0.42 | 2.39 | 0 |
|  | 2.25 | 1.64 | 14.74 | 0.62 | 0.44 | 2.52 | 0 |
|  |  |  |  |  |  |  |  |
| Qdaily_feed | 2.25 | 2.02 | 18.19 | 0.62 | 0.44 | 2.52 | 0 |
|  | 2.33 | 2.02 | 18.19 | 0.59 | 0.42 | 2.43 | 0 |
|  | 2.25 | 1.64 | 14.75 | 0.53 | 0.38 | 2.17 | 0 |
